# Wanga Plant and Voudooism

**Published:** 1878-09

**Authors:** 


### Wanga Plant and Voudooism.

Some months since, noticing a paragraph in a foreign exchange stating that a powerful narcotic plant, unknown to science, was largely used in Hayti as an ordeal poison, the editor of this journal wrote to the government at Washington, asking that inquiries be made. Secretary Evarts very courteously and promptly wrote to the minister at Hayti, requesting an investigation, and the result is so interesting that the letter is here published, with the omission of certain non-essential details.

No. 65.

LEGATION OF THE UNITED STATES,
PORT-AU-PRINCE, HAYTI, June 24, 1878.

HONORABLE WILLIAM M. EVARTS, Secretary of State, Washington, U. S. A.

SIR,—Referring to your No. 12, of January 8 last, I have the honor to state that I have given the subject therein submitted, upon the letter of Professor Wood, due investigation.

The particular plant to which the name of "Wanga" is applied is not known by any one outside of the circle of the high functionaries,--the king, the queen, the papalois, and perhaps some of the more distinguished followers of the Voudoux. There can be no doubt that there is, among other things used by the king and queen, or priest and priestess as they are frequently called, at their initiations, and at other times, as occasion may require, a plant of great narcotic power; and that those who use it have the best knowledge of the character and power of its properties, and how to make application of it so as to accomplish the effects which are desired. The testimony borne in this behalf is abundant and reliable; and it comes to the enquirer in forms and methods both curious and interesting. The more intelligent among the common people furnish entertainment by the hour in telling strange things which they witness as the effect of the manipulation of the plant by the waung, the priest, or papalois. And a like testimony is borne by authors who treat of this subject. In his excellent book entitled "Description de la Partie Française de Saint-Domingue," Moreau, speaking of the Voudoux ceremony of initiation, as translated, says, "The King of the Voudoux makes a great circle with a substance which makes a black mark, and there places the one to be initiated, and puts in his hand a packet of herbs, horse-hair, pieces of iron and other things as disgusting. Afterwards, striking him lightly upon the head with a little battledoor of wood, he (the king) begins singing an African song, which those in the circle repeat in chorus; when the new member sets himself to trembling and dancing; this is what is called 'monter Voudoux.'"

But what the herbs are which the king uses, how they are compounded, what qualities they possess, whether they are the products of this country, and whether he uses more than one, are all matters of conjecture among the uninitiated.

The herb is used on other occasions as well as at initiations. Whenever miracles are to be wrought, the sick healed, the dead brought to life, or any display of power that is superhuman and calculated to strike the masses with awe, is to be made, the herb is used. It is often told with the most profound sincerity and faith, even by those who declare that they do not belong to the Voudoux, that the papalois, a subordinate official of the sect, or even one of them of a still lower official grade, who is moved by what is called the "Lois," can and does resurrect the dead. But the herb always, according to their stories, plays its part in connection with such performances.

The "Lois," as I judge from the statements and explanations of those with whem I have talked on the subject, is a sort of spiritual influence or power, which is sometimes directly bestowed, but more generally inherited. It comes to the child from the parent or grandparent, and when once in the family never forsakes it, but abides forever, descending from mother or father to son or daughter.

The followers of this faith in this country are very numerous, including all grades of social life It is generally believed that the Emperor Soulouque was a member. He certainly did nothing, unlike his successor Geffrard, to prevent its power or its cannibalism. It is well known that the Voudoux are eaters of human flesh, and to secure it do not hesitate to take human life, especially that of small children. With such victims they profess to make sacrifices to their strange god. In connection with these, too, the sacred herb, like the drum and other instruments so constantly used by them, figures conspicuously.

There are several considerations for concluding that the herbs which are used by the Voudoux grow in Hayti.

There is a plant, the product of this country, growing in great abundance, within the reach of those who desire it, whose properties are well known, and which possesses remarkable narcotic power. This plant is often used here on account of its peculiarly narcotic properties, even by the ordinary Haytien not a member of the Voudoux, for medicinal purposes, as well as those that are lascivious and base.

In his work of rare merit, entitled "Flore des Antilles," Descourtilz in the third volume describes the *stramoine épineuse*, or

Datura, and states, in connection with its natural history, that people believe in San Domingo, as he is assured by Colonel Deveux, that the discovery of its somniferous properties is due to a negro, who used to put an old proprietor to sleep, to enable him to steal his bees.

He also tells the story of certain clever negresses who used the same plant to put the lover whom they did not prefer to sleep, while they stole to the embraces of him who was vanquisher.

The juice of the plant, as is well known, has been used not unfrequently to produce temporary blindness: and persons using it have been examined by the surgeons of the government and pronounced blind and unfit for military service, when, after its influence had passed away, the sight was restored, without the least deleterious effect having been produced upon the optic nerve.

Were one to visit the ordinary family of Hayti, and find himself, from fatigue or fear or anguish of mind, wakeful and restless, it is almost certain—if he let his condition be known—that he would be advised of the soporific effects of the datura, and leaves of the plant would be put under his pillow to make him sleep. Five leaves of this plant are said to be sufficient for ordinary cases, under such circumstances.

There are several varieties of this plant found in Hayti. Descourtilz mentions and describes three. In addition to the one already named, he gives the stramoine larmenteuse and the stramoine cormic, the three being described as toxic and bitter-narcotic. All three, in strange but natural combination, may be used by the king or papalois of the Voudoux. Or it may be the case that these are used in combination with other plants, the properties of which have not been made known to science. For I am advised that only six hundred of the two thousand varieties of plants in this country have been examined, classiffed, and their properties determined and defined. Upon this branch of the subject I have had several interviews with Dr. J.-B. Delroux, who is altogether the most learned and scientific man of this republic. He informs me also that efforts will be made, though necessarily in a very imperfect way, through the chair of botany which has lately been established in connection with the medical college of this city, of which he is the president, to explore, to some extent, this field, which must be full of rich treasures for science.

But we may conclude that the stramoine épineuse is the main ingredient of the herbs used by the Voudoux, from the fact that it possesses the qualities, the properties, which work the result which the devotees of this strange religious fanaticism desire; and in this particular species of the plant these peculiar properties are more largely found than in either of the others. Descourtilz, in describing its properties, says:—

"Others mix them with tobacco. Three grains, pernicious to man as it is said, have a property for fattening hogs by causing them to sleep a great deal."

In speaking of it in another connection, he says:—

"This plant, a native of America, is found in all the sandy fields of Europe, where it is perfectly naturalized. The magicians, or pretended sorcerers of the colonies, procured for their sick, in the use of this plant, that species of voluptuous enthusiasm which made them forget, during some instants, the afflictions which oppressed them."

But it must be recollected that it is in the tropics that this plant grows with its greatest vigor and develops its properties in full power. The same species of plant, known as the "Jamestown weed," was formerly found in the states; and in the early settlement of Ohio and other parts of the West it grew quite thriftily; but not as it does here. Its leaf, blossom and fruit, as well as limb and trunk, show that here its roots find the sweetest and most natural nourishment.

Everything connected with the Voudoux service, the serpent, the herbs, the horse-hair, the pieces of horn, as well as the drum, the song, and the circle, have thrown about them a solemn mystery and are held in their sacred uses and effects as profound secrets. Everything is done, the initiation-oath is given and taken, with this object.

All that is said, therefore, with regard to this subject, as already stated, is said upon conjecture. It may have truth in it; it may not.

With sentiments of high regard, I am, sir,

Your most obedient servant,

JOHN MERCER LANGSTON.

